# Establishing spatially-enabled health registry systems using implicit spatial data pools: case study – Uganda

**DOI:** 10.1186/s12911-019-0949-y

**Published:** 2019-11-08

**Authors:** Augustus Aturinde, Nakasi Rose, Mahdi Farnaghi, Gilbert Maiga, Petter Pilesjö, Ali Mansourian

**Affiliations:** 1 GIS Centre, Department of Physical Geography and Ecosystem Science, Lund University, Sölvegatan 12, 223 62 Lund, Sweden; 2 College of Computing and Information Science, Makerere University, Kampala, Uganda

**Keywords:** Spatially-enabled health registry, SDI, RESTful web services, Spatial epidemiology, Mobile-GIS, Uganda

## Abstract

**Background:**

Spatial epidemiological analyses primarily depend on spatially-indexed medical records. Some countries have devised ways of capturing patient-specific spatial details using ZIP codes, postcodes or personal numbers, which are geocoded. However, for most resource-constrained African countries, the absence of a means to capture patient resident location as well as inexistence of spatial data infrastructures makes capturing of patient-level spatial data unattainable.

**Methods:**

This paper proposes and demonstrates a creative low-cost solution to address the issue. The solution is based on using interoperable web services to capture fine-scale locational information from existing “spatial data pools” and link them to the patients’ information.

**Results:**

Based on a case study in Uganda, the paper presents the idea and develops a prototype for a spatially-enabled health registry system that allows for fine-level spatial epidemiological analyses.

**Conclusion:**

It has been shown and discussed that the proposed solution is feasible for implementation and the collected spatially-indexed data can be used in spatial epidemiological analyses to identify hotspot areas with elevated disease incidence rates, link health outcomes to environmental exposures, and generally improve healthcare planning and provisioning.

## Background

The central paradigm of epidemiology is that disease patterns in populations can be systematically analyzed to understand causes and possible control of diseases. This involves comparisons of differences and similarities in disease patterns over time and between places, to gain new insights about the disease [1]. Given that epidemiology is concerned with disease patterns in human populations as opposed to individuals [2], and that these populations tend to inhabit space in non-homogeneous ways, the resulting disease patterns are often non-homogeneous and spatially-dependent [3].

Spatial heterogeneity in both disease risk and disease incidence at fine-spatial scales is well documented and is driven by genetic, social and environmental factors that subsequently affect exposure and response to infection [4]. As such, most health-related issues such as outbreaks and other epidemiological threats are better understood from a spatial-temporal perspective [5]. This then necessitates the recording of fine-scale spatial details of patients along with other personal data upon hospital admission.

Different countries have devised different mechanisms to enable the capture of fine-scale spatial details of persons. These include the use of postcodes for the UK, ZIP codes for the USA, and personal numbers for Scandinavian countries [4], to mention but a few. These codes and numbers are geocoded and therefore enable the capture of spatial positions of patients at a high spatial resolution level, upon hospital admission. Subsequently, these spatial positions are used in epidemiological analyses to identify where disease incidents are common and identify possible local risk factors involved to help in intervention, prevention, and control.

For most developing and resource-constrained countries, mainly in Africa, however, there are no such systems for home and personal addressing [6–8]. The lack of such ways to enable capture of high resolution personal spatial details forces the healthcare personnel to inevitably aggregate patient data, upon admission, to coarser level administrative units. The implication of this coarse-level aggregation is that fine-scale heterogeneity and the role of local contextual factors may be masked. The masking of such heterogeneity derails intervention and control programmes, especially for infectious diseases, as identification of target foci of transmission and local risk factors are obscured at larger scales [9].

Collection of spatial data is an expensive and time-consuming venture. For sustainability, there is a need for approaches that allow for reusability and sharing of already collected spatial data. Reusability and sharing of spatial data are at the core of spatial data infrastructures (SDI). However, given that traditional establishment of SDIs requires a top-down approach with financing originating from national governments [10], their establishment in resource-constrained countries, such as Uganda, have so far not been very feasible. However, SDIs offer advantages that cannot be ignored.

This study pragmatically addresses this challenge by making use of existing ‘spatial data pools’. Spatial data pools in this study are defined as spatial data that have already been collected by different organizations and for other purposes but can be reused for another purpose – for spatial location of patients in our case. By linking this data to patient information, the patient information is georeferenced. In this paper, we address this idea through developing a prototype system using lightweight web services technologies and mobile-based Geographical Information System (GIS) to create a patient registry system that enables recording of such fine-scale patient spatial data, upon hospital/healthcare centre admissions.

The advantage of our system is that it can be integrated with existing health registry (information) systems such as those present in Uganda to make them spatially enabled. It also addresses the challenge of access to desktop computers at healthcare registries by enabling health workers to use their mobile devices to register patient records that can then be visualized and analyzed on the web, reducing the use of paper-based databases – a situation too common in developing countries, like Uganda.

We thus contend that with the proper use of existing spatial data resources, African societies can be spatially enabled by incorporating spatial data in different national information systems in health, tax, police, etcetera that are developing fast.

### Related studies

A variety of approaches have been used, in different studies, to collect disease/health-related data with fine spatial resolution. However, these approaches (as reviewed in this session) do not provide a solution for sustainable and continued data collection, on extensive scales like at countrywide levels.

Karas [6] proposed the use of Global Positioning System (GPS) technology to capture a patient’s homestead location in areas where the patient’s address may be indicated as: “after crossing the river, climb the third hill on the left”. He thus advocated for a latitude and longitude file system, especially for rural African hospital, in tracking infection outbreaks and enabling spatial-epidemiological analyses. Using the GPS/GIS approach, Tanser and Wilkinson [11] quantified the improvement in access to Tuberculosis care in Hlabisa, South Africa, when the hospital, clinic, community health workers (CHW), and patient locations are known. They found that by using key locations, the mean distance from patient homestead to point of care (hospital, clinic or CHW) reduced from 29.6 km to just 1.9 km. Similarly, Dwolatzky et al. [12] while studying patient adherence to TB medication in Johannesburg, South Africa, used handheld computing devices (personal digital assistants – PDA) with GPS capabilities to trace the location of patients. By comparing the time taken while using the device, and while not, they found that using PDA/GPS devices reduced the locating time by up to 50%.

Whereas these studies showed considerable success in the use of GPS and PDAs, it must be appreciated that they were used in small towns, where the mapping of individual patients is possible. Scaling up of this approach to a regional or national level would be too expensive to sustain. Consequently, the conference of the African Federation of Emergency Medicine recommended for the use of existing mobile technology to optimally solve patient location problems in Africa [7].

The use of mobile technology in the provision of medical care is not new. Working from the knowledge that Dementia patients are at a higher risk of wandering and getting lost due to a decline in cognitive functioning [13], Huang et al. [14] implemented a pilot program that sends the GPS coordinates of the patient, using a passer-by phone, to service centre personnel, using near-field communication tags embedded in the patient’s wristband. In a similar approach, Mendoza et al. [15] proposed tracking and locating of patients with Alzheimer’s disease in a nursing home by the use of a wearable tracking device. The device continuously transmits the patient location and sends notification messages to a monitoring database whenever the patient wanders beyond the designated limits. Whereas these are good approaches, they are very case-specific and work best for small special groups of patients. Also, the need for programmable devices makes costs unbearable and makes the approach impractical especially when large numbers of patients, in a resource-constrained setting, spread all over a large area are involved.

Fornace et al. [8] used android tablet-based applications to geo-locate malaria patients in rural Philippines and Indonesia. Their study provided a way of obtaining high-resolution spatial data in resource-constrained societies with poor internet connectivity, even though their approach is affected by the same scalability issues identified earlier. Additionally, their approach has an inherent requirement for retrospective collection of patient spatial data after patients had been discharged, making it laborious and prone to missing some people. Finally, in the face of another disease, there would be a need for another fieldwork to collect patient location data hence no reusability of the already recorded spatial data.

The challenges in these previous studies can be summarised as (1) retrospective collection of patient location after hospital discharge is both laborious and may miss out some patients; (2) it is challenging to scale up retrospective collection of patient location details when a large area and a large number of patients is concerned; and (3) the patient registry system at the healthcare units is not improved. These three challenges to health informatics, i.e. completeness of records, scalability, and improvement of the existing registry system, are addressed in this study.

## Methods

Currently, there are three main health registry systems implemented by the Ministry of Health (MoH) in Uganda.

- OpenMRS – globally adopted open source electronic health registry, used mainly for HIV/AIDS reporting in Uganda [16].
- mTrac – an SMS-based health system originally used to report real-time stocks status of malaria drugs and vaccine at health facilities, but was modified to handle reporting of disease admissions too [17].
- eHMIS-DHIS2 – a community-based aggregation system that scales from the lowest level to the national level [18].

eHMIS-DHIS2 (hereinafter called DHIS2) is currently the official health reporting system in Uganda. The system provides monthly summaries of health status at the district level based on the data from both mTrac and OpenMRS platforms. These monthly aggregates are then transmitted to the national level for archiving, summarising and maintenance. The inherent aggregate architecture in both mTrac and DHIS2 does not allow them to capture personal-level spatial details. Also, whereas OpenMRS records personal details, it does not capture the patient’s residential location along with other patient details, upon hospital admission or consultation.

While there is no addressing system in Uganda to collect or geo-reference patient residential locations, the National Water and Sewerage Corporation (NWSC), a central body responsible for water distribution in Uganda, has a database including the geo-coordinates of water meters which are identified through unique water meter numbers. This database is updated regularly with the development of the water network in Uganda. Also, all connected households have access to their water meter numbers, which is written on their monthly water invoices.

The suggested idea by this study is that by asking the patients to report their household NWSC meter numbers along with other personal details upon hospital admission, the spatial locations of their residences could be uniquely identified. Subsequently, they can be used not only in e-health services delivery but also in epidemiological analyses, intervention, and control. This way, the georeferenced meter numbers act as our spatial data pool. Enablement of this capability requires a linkage between the NWSC database and the health registry database in an interoperable way. Also, tools/systems are needed for the digital collection and registry of patients’ information.

### The overall architecture of the system

**Fig. 1:**
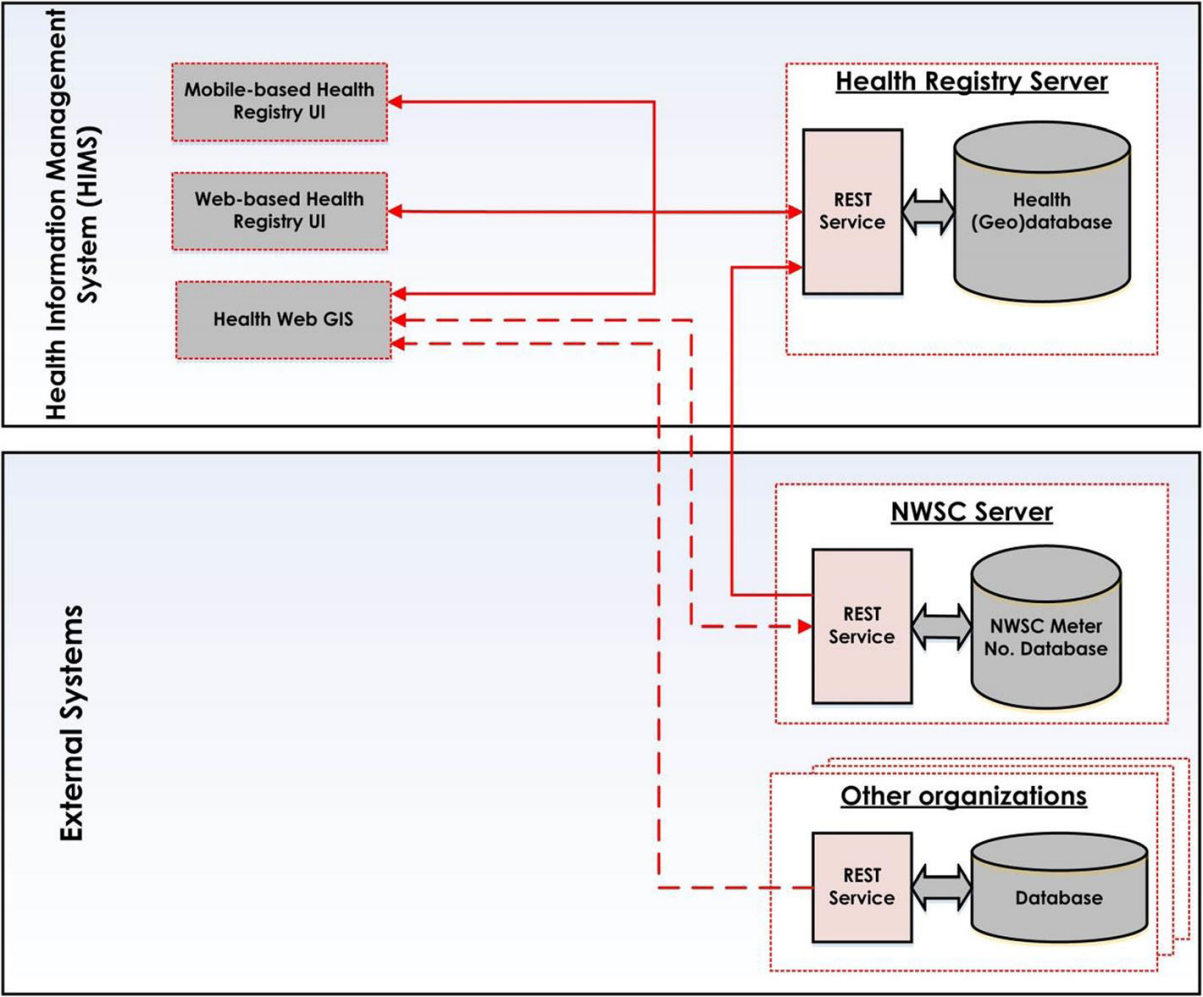
Schematic representation of the proposed spatially-enabled health registry system

Figure 1 shows the overall architecture of a system for a spatially-enabled health registry and how it enables eventual spatial epidemiological analyses. The system is made up of the following components.

- *Mobile-based health registry UI* (user interface) and *web-based health registry UI* are used by medical personnel of healthcare centres, to register patients’ admission details and patients’ residential location. The geo-coordinates of patients’ residence are either retrieved from the NWSC database through REST services or pined on the map using the health registry UI components.
- *Health registry server* provides the ability to save in and retrieve health registry data from a (Geo) database through a REST Service.
- *NWSC server* provides the ability to access the water meter numbers and their respective geo-coordinates from the NWSC database through a REST service.
- *Health Web GIS* enables the healthcare personnel to analyse the admission data collected by the system as well as the data from other organisations that are published as REST Services (e). These analyses can be used to answer specific spatial epidemiological research questions.
- Other organizations can participate in this system by publishing their data through REST Services. Such data can then be used by the Health Web GIS component for contextual epidemiological analysis.

At any hospital or healthcare centre within the country, a mobile application providing the *mobile-based health registry UI* (a) is installed on handheld devices of medical personnel responsible for patient data recording. Alternatively, *web-based health registry UI* (a) on their computers can be used. The medical staffs, including nurses and doctors, use these UIs for registering patient’s information including disease diagnoses and their residential locations.

To record patient residential locations, patients are asked to specify their water meter numbers (provided and maintained by NWSC). The UI components also have a background map, with satellite imagery, by which the approximate location of the patients’ houses can be navigated and marked for those without water meter numbers (possibly not yet connected to the water network) or are unable to access them for some other reason. The registered information is sent to and stored in the health registry (geo)database. In the health registry (geo) database (b), a patient’s information is geo-coded by using geo-coordinates received from the NWSC database (c) (with meter number as the unique identity).

A Health Web GIS system (d), with spatial analysis functions including those needed for spatial epidemiology, has access to the health registry database (b) and other databases (e) (e.g. environmental data from related organisations). So, spatial epidemiology analysis is possible to detect disease hotspots, outbreaks, monitor the progress of diseases in space and time, prepare prevention plans, etc.

### Development and implementation

The *mobile-based health registry UI* was developed as an android app by Java programming language. JavaScript programming languages, Cascading Style Sheets (CSS), and HyperText Markup Language (HTML) were used to develop the *web-based health registry UI* as well as the *Health registry Web GIS*. In order to provide mapping functionalities in the web applications, the Leaflet library (https://leafletjs.com/) was exploited. To develop the web services, two frameworks: Service Oriented Architecture Protocol (SOAP) and REpresentational State Transfer (REST) are commonly used. However, SOAP has a heavyweight message payload thus not very favourable for resource-constrained mobile devices [19]. Subsequently, the REST web service framework was used in our study as its messages have a lightweight payload hence more suitable for wireless and cellular connectivity networks synonymous with mobile devices [19]. The REST services were developed in Java programming language using oracle JAX-RS.

The system was implemented as a prototype and presented to the Ministry of Health officials. The idea was welcomed as a prospective candidate to complement patient registry systems in Uganda since it covers the gap in existing systems which do not record the absolute spatial location of patients’ homes. Discussions about the adoption of this system are ongoing and decisions will have to be made at higher levels of the Ministry of Health.

## Results

Below we describe a scenario where a patient visits a healthcare unit and how the system is used to capture the patient details, including the patient’s home location.

**Fig. 2:**
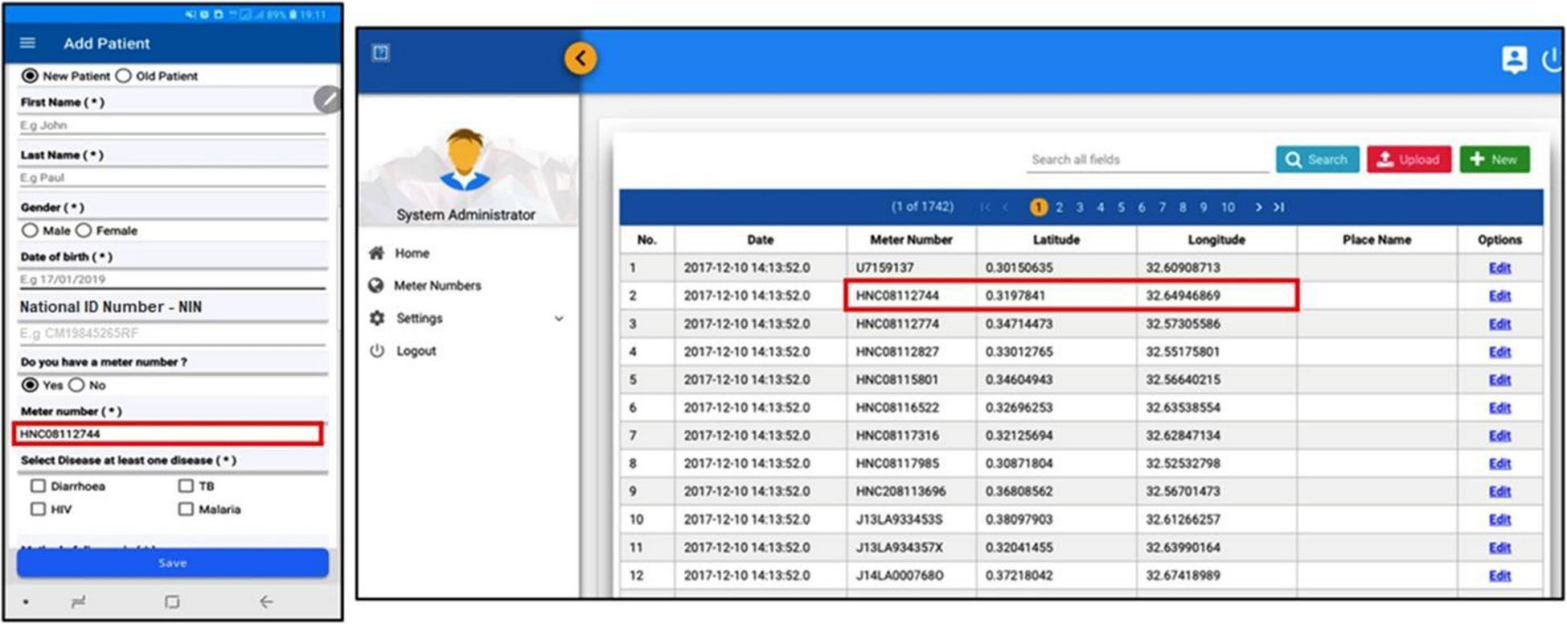
Mobile App UI for a patient with a METER NUMBER and their geo-coordinates from the NWSC database

When a patient visits a healthcare centre, the healthcare provider (doctor, nurse, etc.) collects and registers the patient’s personal information and the disease diagnosis through the *mobile-based* or *web-based health registry UIs*. To avoid duplication of patient records, the National Identity Number (NIN) is required for every patient. As mentioned before, the water meter number of the patient is also asked to geocode a patient’s residence location. Figure 2 shows the interface of the mobile app, with its corresponding geo-coordinates as synched from the NWSC database.

**Fig. 3:**
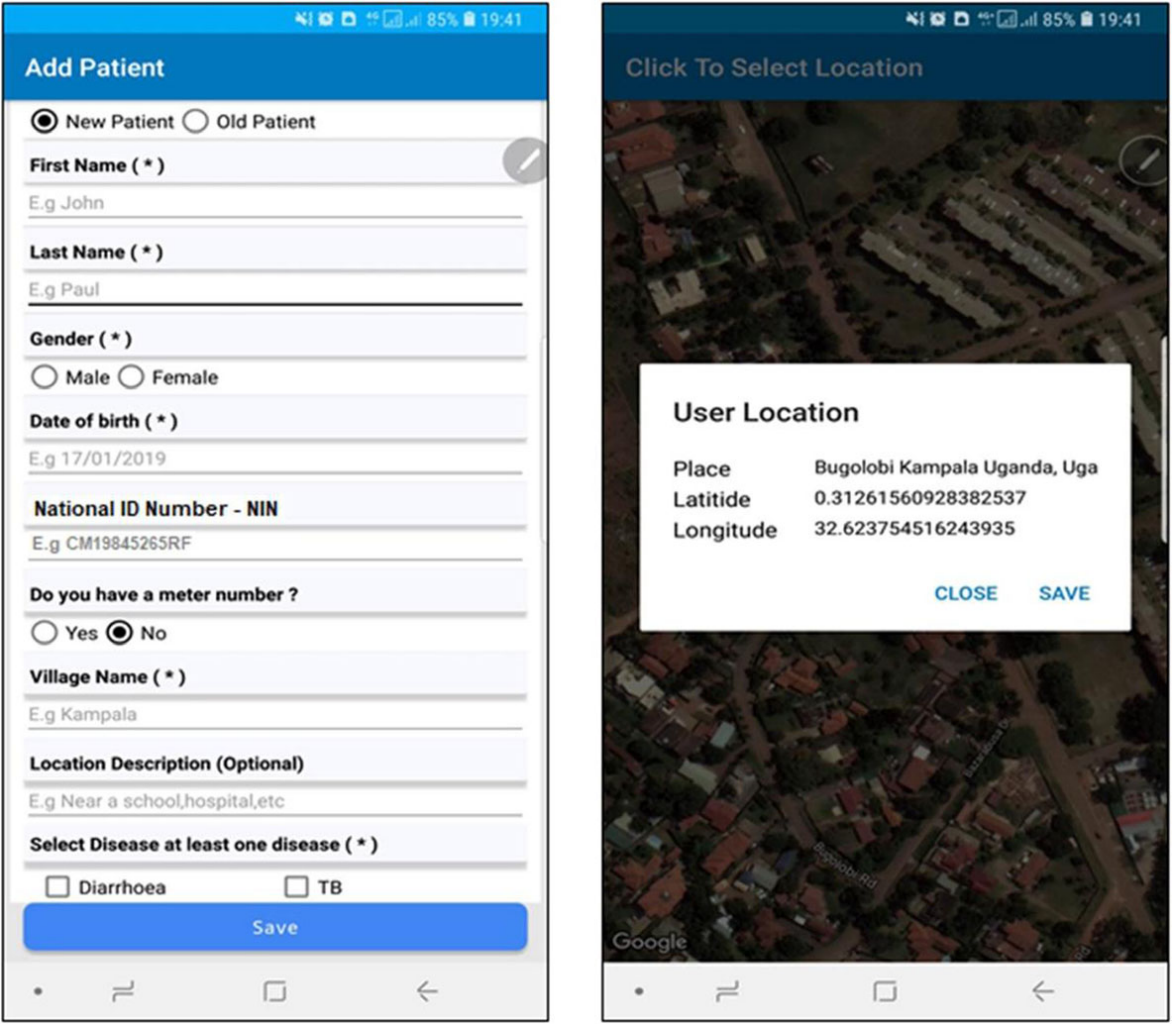
Patient without a meter number and geo-coordinates collected using a background high resolution map

If a patient has no meter number, a background map with satellite imagery is launched (Fig. 3) with navigation capabilities to navigate to the patient’s home. The same high resolution background map would be used in instances where the patient forgets his meter number or is unable to provide it for some other reason. Upon identifying the home, a single click on the home retrieves its coordinates, prompting the user to save the coordinates against the patient’s record. All the information gets stored in the health registry database upon saving.

The coordinates are therefore either retrieved from the geocoded meter numbers or from the background satellite imagery map. All the information gets stored in the health registry database.

Through the Health Web GIS interface, the location of patients and diseases can be retrieved and used for analysis. Using this interface, one may find the spread of a particular disease by viewing and analysing the recorded data or may integrate the patient and disease data to other data to perform more specialized spatial analyses. Below we illustrate the possible uses of the system using an example.

**Fig. 4:**
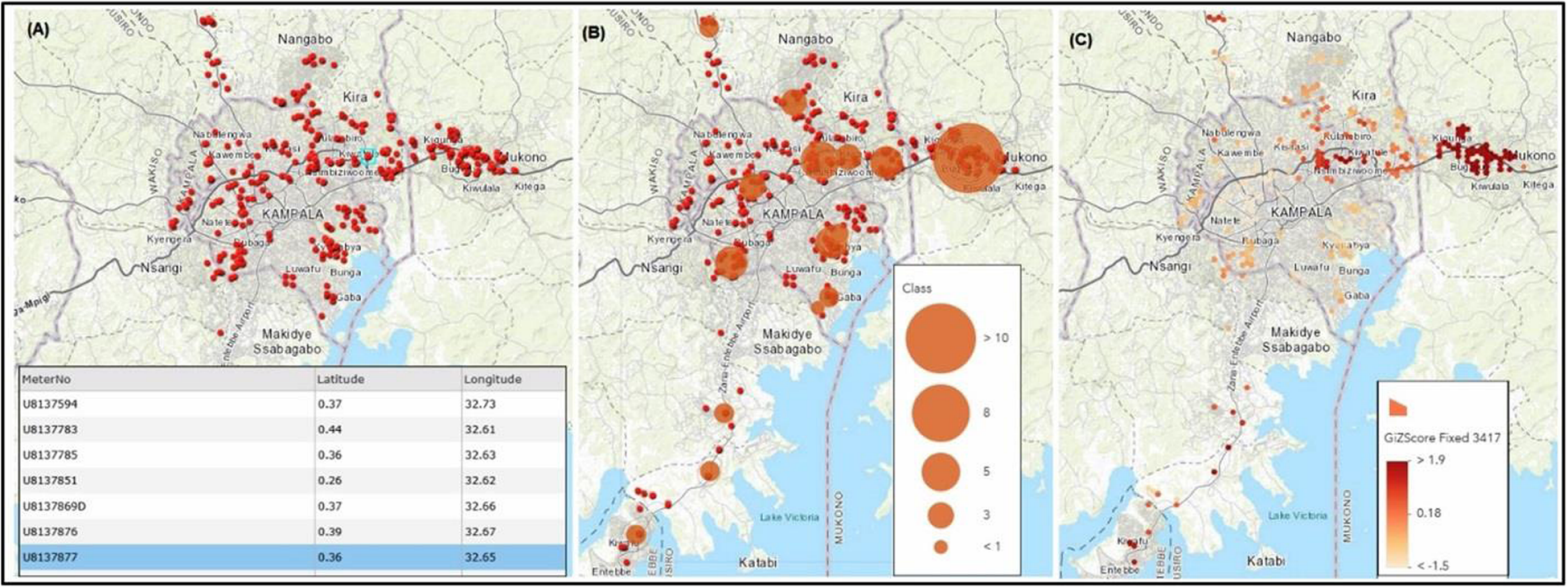
Some of the possible analyses with geocoded patient records: Incidence (**a**), Density Clustering (**b**), and Hotspot Analysis (**c**). (Map generated from sample collected data and produced by the authors. The background map is from ArcGIS-Online)

Figure 4 illustrates a snippet of what can be done with the recorded patient information. In Fig. 4 (a), the incidence data are plotted to show where the incidences are spatially located. By applying cluster analysis, one can study where the incidences could be high and where they are low. In (B), we use point density to highlight areas with more admissions. This results in the visualisation of potential clusters on the map. To investigate which of these potential clusters constitute a hotspot or cold spot, hotspot analysis may be applied. Fig. 4 (c) shows the outputs of hotspot analysis. It shows that among the identified density clusters, only a few are statistically significant (arising not by chance). There is a very pronounced significant hotspot as shown by the GiZscore, on the far right of Fig. 4 (c) and some noticeable ones to the left of this far right hotspot.

This fine detail spatial analysis could not be possible with the current health registry systems. In terms of directing intervention, the healthcare professionals and policymakers are better informed with this kind of tool, not only in disease surveillance but also in associative analyses to evaluate the environmental factors at play in specific disease scenarios.

## Discussion

Williamson et al. [20] described a spatially enabled society as an emerging cultural and governance revolution offered by spatial information technology and spatially equipped citizenry that change the way economies, people and environment are organised and managed for the better. Thus, spatial enablement, itself is a consequence of the realisation of SDI promises through developing spatial information products, smarter delivery of services, improved risk management and better macroeconomic decision making.

The establishment of SDIs is still largely dependent on national governments’ initiatives [21] especially through the establishment of complete national land cadastre systems [20, 22], even when synergistic two-sided involvement of public institutions and entrepreneurs in SDIs is currently being encouraged [23]. The overarching benefit of such SDI establishments lies in the better national management of both spatial information and information organised according to location. However, due to the lack of a means to capture spatial data as well as the inexistence of SDIs in most of the resource-constrained African countries, SDIs and hence spatial enablement of society have not been achievable in most parts of Africa. For example, a country like Uganda does not even have a street addressing system and has an incomplete cadastre.

Our study shows that by using creative approaches, operational systems can be developed to achieve a spatially enabled society in many aspects, as we have done in this study using the case of healthcare registry. Wallace [24] illustrated that spatial enablement has two stages: the first stage involves utilising of imagery to answer the basic question of “where am I?” while the second stage involves linking all data with a geocoded reference to allow for spatial analysis and spatial decision making.

In this study, we utilized spatially referenced water utility data and RESTful web services to implement a prototype of a spatially enabled health registry system in Uganda. Our system achieves the enablement stages outlined by Wallace [24] by (1) using publically available google maps satellite imagery services to identify patient residencies in instances where the patients do not have the meter numbers, and (2) using geo-referenced water utility meter numbers from NWSC to enable geocoding of patient-level records upon hospital admission, using RESTful web services. We used RESTful web services for they have a lightweight message load and is more adapted for mobile network connectivity [19, 25]. This kind of system can be generalised to other (East African) countries that have such spatially referenced utility data like Kenya [26], Tanzania and Rwanda whose utility sector is relatively similar to the Ugandan sector.

Our system, to the best of our knowledge, is the first spatially enabled health registry system in Uganda. Being a digital system, our system like other existing health information registry systems (OpenMRS, mTrac, DHIS2) helps to get rid of paper databases that are still common in Uganda. Additionally, our system is easy to integrate with existing systems, especially the OpenMRS [16] that inherently records individual patient-level details, as opposed to aggregate systems like mTrac [17] or DHIS2 [18]. Finally and more importantly, our system allows for the collection of spatially referenced medical records that can be used in spatial epidemiological analyses and health planning as we illustrate in Fig. 4. The proposed solution for spatial-enablement of health registry is not expensive to implement, especially in comparison with a real implementation of an SDI.

In terms of enabling the capturing of geographical coordinates of patient homes, our study compares to systems by Tanser and Wilkinson [11], Dwolatzky et al. [12] and Fornace et al. [8]. However, unlike these previous studies, our study achieves this but also has capabilities of enabling existing registry systems at the healthcare units. It, subsequently, eliminates the need to capture the patient home location retrospectively after being discharged from the hospital.

The advantages of such a system that enables the recording of the spatial location of patients’ homes are that it makes tracking of infectious diseases, identification of health trends, identification of disease clusters and linking of environmental exposure to health outcomes, possible. It also improves service delivery as medication can be delivered on doorsteps, especially necessary for diseases (mainly terminal illnesses) that are better managed from home.

Whereas our study achieves what it set out to achieve, we acknowledge some challenges that were either encountered in the study or challenges that could influence the adoption of such a system. For example, there were some difficulties in convincing NWSC to share their meter number data, as the solution was to help the healthcare industry, not the water industry. We solved this issue by convincing NWSC on how their help can contribute to increasing the health and the quality of life in Uganda. The system also requires some preliminary training of medical personnel to use the system especially map reading and navigation when the patient does not have a meter number (possibly not yet connected to the national water network). The time required to navigate to patients’ homes might slow down the registration process and decrease efficiency, especially for novice users. However, from a cost-benefit perspective, the benefits of collecting positional information of patients are more than the cost/time spent. Additionally, we are convinced that this system is extendable to most developing countries whose spatial data infrastructure situation is similar to Uganda’s (incomplete digital cadastre, no geocoded street names and no postcodes). However, the lack of any geocoded data could be a limitation to applying the suggested idea in those countries. Finally, the patients in such a system cooperatively give their home location details, when there are enforcing laws, like laws in many European countries. Privacy and confidentiality laws are needed in Uganda to hinder the distribution of individual patient’s records (for example only aggregated data may be analysed and distributed [4]) and also to satisfy ethical constraints.

## Conclusions

Recording of patient residential locations normally requires the use of established country-specific spatial data infrastructures that are inexistent in most developing countries. By using geo-coded data already collected for utility services provision, RESTful web services and mobile technology, our study provides a valuable possible improvement to existing electronic health registry systems that enable them to be spatially-enabled hence increasing their return on investment. The return on investment is in the form of extra capabilities that spatially-enabled health registry systems have over currently existing ones such as identification of areas with elevated disease incidence rates, identification disease trends across space and time, aiding targeted intervention as well as linking environmental exposures to health outcomes. Finally, by explicitly capturing patients’ residential locations, such services as location-aware emergency and prescription delivery can be enabled thereby improving general healthcare planning and provisioning.

## Data Availability

The datasets generated and/or analysed during the current study are not publicly available due to integrity and legal reasons but are available from the corresponding author on reasonable request.

## Abbreviations

AIDS: Acquired Immune Deficiency Syndrome
CHW: Community Health Workers
CSS: Cascading Style Sheets
GIS: Geographical Information Systems
GPS: Global Positioning System
HIV: Human Immunodeficiency Virus
HTML: HyperText Markup Language
NWSC: National Water and Sewerage Corporation
REST: REpresentative State Transfer
SDI: Spatial Data Infrastructure
SOAP: Service Oriented Architecture Protocol
UI: User Interface
